# Digital Media for Health Outcomes: Evaluation Study of a Massive Online Open Course

**DOI:** 10.2196/85016

**Published:** 2026-06-25

**Authors:** Hannah Melchinger, Sarah Christie, Abigail Titus, Alyssa Cruz, Chelsey Lepage, Tony Foleno, Colleen Thompson-Kuhn, Adam Terefe Abebe, G. Nekerwon Gweh, Donewell Bangure, Stephen Maina, Judy Mwangi, Peninah Thande, Maike Winters, Amyn A Malik, Aleksandra Kuzmanovic, Surangani Abeyesekera, Sara Epperson, Whittney Tom, Kylie Holmes, Angus Thomson, Saad B Omer

**Affiliations:** 1 Peter O'Donnell Jr. School of Public Health University of Texas Southwestern Medical Center Dallas, TX United States; 2 Yale School of Medicine New Haven, CT United States; 3 Poorvu Center for Teaching and Learning Yale University New Haven, CT United States; 4 Yale Institute for Global Health Yale University New Haven, CT United States; 5 Irimi Thiers France; 6 Ad Council New York, NY United States; 7 Africa Centres for Disease Control and Prevention (Africa CDC) Addis Ababa, Addis Ababa Ethiopia; 8 Population Services International Nairobi Kenya; 9 World Health Organization Geneva Switzerland; 10 United Nations Children's Fund (UNICEF) New York, NY United States; 11 The Permanente Medical Group Oakland, CA United States; 12 (Formerly: 2018-2023) Meta Platforms, Inc. Menlo Park, CA United States

**Keywords:** digital health, health campaign, online course, massive online open course, MOOCs, public health campaign, social media

## Abstract

**Background:**

Digital media has emerged as a vital tool for the rapid dissemination of public health information to vast audiences. During the COVID-19 pandemic, social media-based health communication kept audiences informed, encouraged health-seeking behaviors, and helped address rampant misinformation. However, the impact of these digital communications was limited by a lack of training in leveraging online advertising tools, behavioral insights, and marketing practices to target, test, and scale social behavioral communications for health. The COVID-19 pandemic highlighted an urgent need for greater capacity among public health organizations to rapidly develop and deploy impactful, evidence-based digital campaigns.

**Objective:**

This study aimed to evaluate changes in learner confidence and perceived ability to implement key skills after taking a massive online open course on digital campaign creation.

**Methods:**

*Digital Media for Health Outcomes* was launched in May 2023 to train public health practitioners in all steps of campaign development, from evidence and insights collection to outcome evaluation. In April 2025, course performance was evaluated using a pre-post survey design. Learners were asked to complete identical surveys before and after completing the course. Question topics included basic demographic information and learners’ confidence and experience in applying key campaign development skills. Learners were also asked to rate their confidence in using digital media to drive health outcomes before and after taking the course, and their perceived importance, ease, likelihood, and effectiveness of doing so. Proportions and 95% confidence intervals were calculated for confidence questions, and proportion changes were compared between the baseline and endline surveys. All analyses were conducted using Stata (Special Edition 18; StataCorp).

**Results:**

As of April 2025, the course had enrolled 14,170 individuals in 9 language versions, representing over 160 unique countries from all global regions. A total of 4392 learners completed the baseline survey, and 634 completed the course and endline survey. Most course completers came from Chile (240/634, 38%) and the United States (69/634, 11%). Learners who completed the course reported significantly higher confidence (+18%, 95% CI 17%-20%) in their perceived ability to use digital media to drive health outcomes and in applying skills associated with effective campaign development, including leveraging behavioral insights (+16%, 95% CI 14%-18%) and evaluating campaign outcomes (+28%, 95% CI 26%-29%). Overall, 92% (583/634) of course completers felt that the course had met most, all, or exceeded their expectations, and 85% (539/634) indicated that they would recommend the course to a colleague.

**Conclusions:**

Performance evaluation of the *Digital Media for Health Outcomes* course suggests the potential of an expert-led, practice-based massive open online course to improve confidence and skill level in leveraging digital media for effective health communication among public health professionals. Further capacity strengthening is needed to encourage skill application.

## Introduction

Communication is an essential component of public health practice. During the COVID-19 pandemic, the need for effective public health communication became critically apparent. The conflation of emergent and evolving information and public guidance around disease prevention and spread—in addition to the interruption of routine health services, introduction of new vaccines, discovery of information vacuums, and explosion of mis- and disinformation on and offline—eroded public trust in health organizations and messages. Including the United Nations International Children’s Emergency Fund (UNICEF), the World Health Organization (WHO), and the United States Centers for Disease Control and Prevention (CDC) on infection prevention, management, and treatment. In the years since the pandemic, social media–based campaigns have become increasingly important in combatting the ongoing “infodemic,” characterized by the overload of online information, including mis- and disinformation. [1,2].

The COVID-19 pandemic also highlighted the potential of digital communication—particularly social media—as a cost-effective way to reach vast audiences with relevant, timely, and targeted public health updates. As of 2024, over 94% of global internet users use social media monthly, with platforms like Facebook (Meta; 3 billion users), YouTube (Google; 2.5 billion), Instagram (Meta; 2 billion), WhatsApp (Meta; 2 billion), and TikTok (ByteDance; 1.9 billion) leading global engagement [3-5]. During the pandemic, health professionals and organizations began using or increased their use of these platforms to reach audiences with updates on infection prevalence and mitigation strategies; several studies have found that information shared about COVID-19 via social media influenced audiences’ knowledge, attitudes, and self-reported behavior [6-8]. However, few studies have assessed the effect of offline campaigns on online behavior [9].

Effective health communication strategies must be grounded in behavioral evidence and tailored for context. A recent systematic review of insights-based interventions described several effective strategies to shift health attitudes and behaviors, among them message framing, educational campaigns to raise awareness and knowledge, and the use of trusted messengers [10]. These interventions factor in local context, cultural considerations, communication preferences, behavioral evidence, and proven behavior change levers to effectively address concerns and questions among intended audiences [11-14]. Overall, evidence-based behavior change communication relies on a keen understanding of intended audiences, their values, and their concerns to provide trustworthy, impactful health messages.

To effectively communicate and meaningfully influence attitudes and behaviors via digital media, public health communicators need to apply social and behavioral change communication (SBCC) principles to digital communication campaigns but often lack the requisite skill set. In collaboration with the Ad Council, African Union | Africa CDC, Meta, Population Services International, UNICEF, the Yale Institute for Global Health, and the Poorvu Center for Teaching and Learning at Yale University, we designed an online course to lead practitioners through the process of developing and evaluating a digital health SBCC campaign. The *Digital Media for Health Outcomes* (DMHO) course draws on the evidence and real-life experience of diverse partner organizations to deliver accessible, practical, and effective training in digital social and behavior change communication for public health. Since its public launch in May 2023, the DMHO course has enrolled more than 14,170 learners from 162 countries and certified over 630 public health communicators. Here we present lessons learned from course evaluation data and offer recommendations for strengthening capacity in public health communication through training and online education programs.

## Methods

### Course Development

In 2021, as part of its global response to the COVID-19 pandemic, Meta spearheaded a pilot course with UNICEF, Capulet, and other partners to support public health agencies and nongovernmental organizations in developing effective health communications for the digital space. In 2022, the DMHO course was proposed to build on this work, and the course syllabus was developed collaboratively during an all-partner meeting in August 2022.

**Figure 1:**
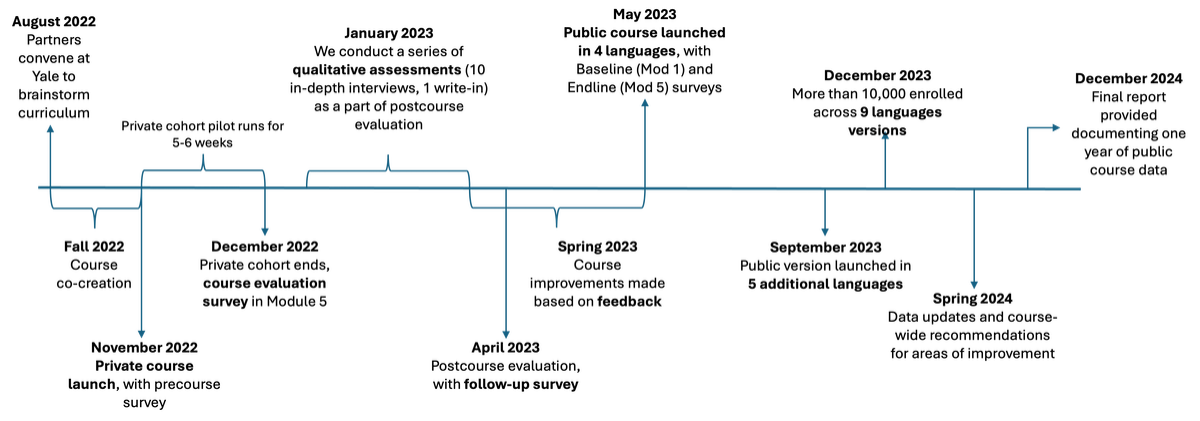
Development and evaluation timeline for the Digital Media for Health Outcomes course.

Delivered over 5 modules, the course was designed to support public health agencies and organizations in creating effective digital health behavior change campaigns. From November to December 2022, we piloted the course with a private cohort of 100+ health and communication practitioners from partner organizations over a 6-week period to test, validate, and strengthen content and delivery. The course was launched for public consumption in May 2023. As a part of course maintenance and improvement, we performed regular course evaluations and updates (Figure 1).

### Course Structure

The course consisted of the following 5 modules (Table 1). Each module consisted of instructional videos, in-video quizzes, readings, and expert case studies. Progress was assessed through a simple, relevant homework assignment graded through the Coursera peer review system, by which course learners were able to leave feedback on each other’s work. The course was hosted on Coursera, an online learning platform offering free and tiered payment access [15]. The course was available in all 9 United Nations and African Union languages, including Arabic, Bahasa Indonesia, Chinese, English, French, Portuguese, Russian, Spanish, and Swahili. Translations of all course content were done by WeTranslate, a professional translation agency. The course content was available for free; if learners wanted to receive a Yale certificate upon course completion, they could pay for a version of the course costing approximately US $50, payable either by individual learners, learner organization, or a Coursera Plus subscription. However, all those who enrolled could apply for Financial Aid and receive the certificate for free.

**Table 1:** Course modules.

Module	Title	Description
1	Behavioral Insights as a Foundation	Introduces learners to the concept of behavioral insights and demonstrates how these insights can be identified to inform evidence-based, targeted health communications. Africa CDC^a^ presents a case study on Ebola response strategies.
2	Crafting Your Communication Strategy	Learners identify their target audiences and craft a strategic brief, following best practices shared by experts. The Ad Council presents a field perspective on a successful lung cancer screening campaign, Saved by the Scan.
3	Designing for Context: Messaging and Creative that Resonate	Dives into the best practices and key considerations for creating impactful and visually appealing creative assets, with messaging that is meaningful to the target audience. The Ad Council presents a field perspective on creative best practices, while UNICEF^b^ highlights vaccine-specific messaging strategies.
4	Tactics for Digital Media and Campaign Implementation	Discusses the practical steps for launching a campaign on social media, focused on best practices for different platforms and metrics of evaluation with a case study from Population Services International.
5	Metrics that Matter: Understanding Impact	Describes several ways to assess health outcomes in response to health communication campaigns to understand the impact of the work. Case studies are presented by the World Health Organization and Yale Institute for Global Health.

^a^CDC: Centers for Disease Control and Prevention.

^b^UNICEF: United Nations International Children’s Emergency Fund.

### Course Pretest and Launch

The course was first launched for a pilot cohort of public health practitioners from November 2022 to December 2022. Learners, most of whom were UNICEF, WHO, or Ministry of Health employees, were invited based on course partner recommendations to participate in a 6-week, guided version of the course, and asked to provide in-depth feedback on course elements and overall flow. The private cohort included 107 learners representing 42 countries and 55 unique organizations; 83% (88/107) completed the course and submitted postcourse evaluation surveys. Furthermore, 10 completers were randomly selected to participate in individual postcourse interviews, conducted online by the Yale Institute for Global Health and lasting approximately 30-45 minutes. The results of these evaluations were used to update the course for public launch. A public version of the course was officially launched in English, Arabic, French, and Spanish in May 2023. Versions in the additional 5 languages were launched in September 2023.

### Survey Development

The course was evaluated using a pre-post survey design hosted on Qualtrics [16]. At the start of the course (module 1), learners were asked to complete a confidential survey (referred to as the “baseline survey”) to establish their baseline experience in using digital media to promote health outcomes. At the end of the course, learners were asked to complete a similar survey (referred to as the “endline survey”), which also asks them to reflect on elements of the course, what they liked, and what they did not. Survey questions were validated during the pilot version of the course and are available in Multimedia Appendix 1. Survey translations (from English to other languages) were done using Qualtrics’ built-in translation software and verified by knowledgeable team members using DeepL, an online translation program recognized for accuracy. All translations were carefully reviewed for consistency with the English version before survey launch. Within the survey, participants were able to toggle between languages as desired.

Both surveys asked learners a series of demographic questions, including what type of organization they worked for and where their work was based. In both surveys, key performance indicators (KPIs) included learners’ confidence and experience in applying six fundamental campaign skills including (1) understanding and identifying behavioral insights, (2) identifying a target audience online, (3) using best practices in designing creatives, (4) planning a digital communication strategy to drive health outcomes, (5) using practical tactics to implement the communication strategy, and (6) evaluating campaign outcomes. Learners were also asked to rank their confidence from low to high (0-10), with scores of 0-5 categorized as low confidence and scores of 6-10 categorized as high confidence. Similarly, learners were asked to report their perceived confidence in and importance of, ease of, likelihood to, and effectiveness of using digital media to drive health outcomes.

### Statistical Analysis

Data presented in this study were collected from May 2023 to April 2025. All data were self-reported. Proportions and 95% CIs were calculated for all confidence questions, and proportion change was compared between baseline and endline surveys. Incomplete surveys were removed before starting the analysis.

We also conducted a subanalysis of respondents for whom we could link baseline and endline survey results. Response datasets were merged by respondent age, gender, and current organization, provided in the baseline and endline surveys. Demographics and KPIs were assessed as in the main analysis. Deidentified data from both surveys were analyzed using STATA (StataCorp).

### Ethical Considerations

This analysis was classified as a program evaluation (a type of nonhuman participant research) by the Institutional Review Board at the University of Texas Southwestern Medical Center. As such, the study was approved as nonregulated research (STU-2024-0962). Elements of this report were included based on the Western Michigan University Checklist for Program Evaluation Reporting Content. Participants received no compensation for participation. The first page of both surveys advised participants that their participation was entirely voluntary and that their responses would be kept confidential and used to evaluate course performance over time. Respondents provided their consent to participate by proceeding to the next page of the survey.

## Results

### Baseline Learner Demographics

**Figure 2:**
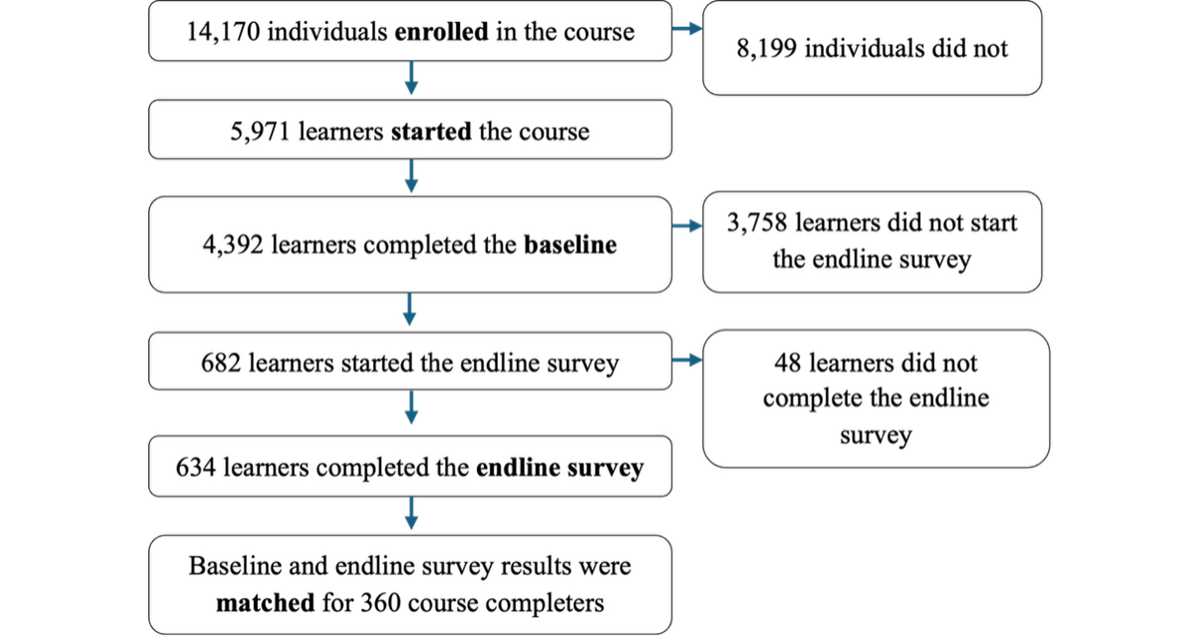
Participant flow diagram.

As of April 2025, a total of 14,170 individuals were enrolled in the DMHO course, and 5971 learners had started the course. A participant flow diagram is presented in Figure 2.

**Figure 3:**
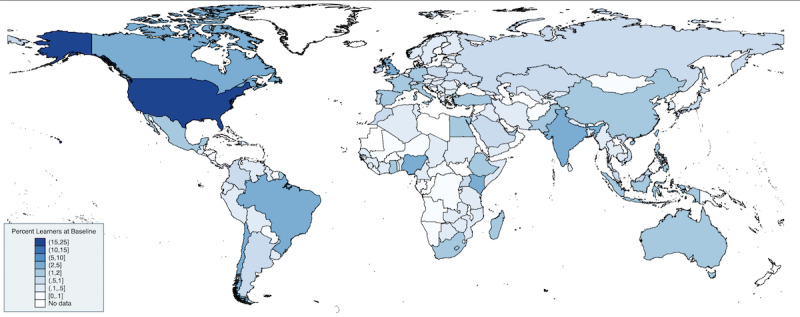
Countries represented by Digital Media for Health Outcomes course learners at baseline, April 2025 (n=4392).

Of these, 4329 (73%) completed the baseline survey (Table 2). The top 5 language versions among learners were English (3394/4329, 78%), Spanish (390/4329, 9%), French (211/4329, 5%), Arabic (116/4329, 3%), and Russian (89/4329, 2%). Most learners identified as women (3010/4329, 69%), were older than 25 years (1974/2464, 80%), and had received at least a bachelor’s degree (3519/4329, 81%). Many learners worked at academic institutions (505/4329, 12%), ministries of health and government agencies (447/4329, 10%), and in the private sector (600/4329, 14%). Several learners were university students (427/4329, 10%). Geographically, learners represented 162 countries and all UNICEF regions. The five countries with the greatest shares of learners were the United States (986/4329, 22%), Chile (208/4329, 5%), India (196/4329, 5%), Canada (153/4329, 4%), and Nigeria (140/4329, 3%; Figure 3). Regionally, learners were predominantly from North America (1121/4311, 26%), Latin America and the Caribbean (597/4311, 14%), and Western Europe (582/4311, 14%).

**Table 2:** Learner demographics for the Digital Media for Health Outcomes course at baseline (April 2025).

Demographic		Baseline survey results, n (%)
**Survey language (N=4329)**		
	Arabic	116 (2.7)
	Bahasa Indonesian	16 (0.4)
	Chinese	33 (0.8)
	English	3394 (78)
	French	211 (4.9)
	Portuguese	80 (1.9)
	Russian	89 (2.1)
	Spanish	390 (9)
	Swahili	0 (0)
**Gender (N=4329)**		
	Men	1210 (28)
	Women	3010 (70)
	Other (nonbinary, self-describing, prefer not to say)	109 (2.5)
**Age (y; n=2464)**		
	13-17	40 (1.6)
	18-24	451 (18)
	25-34	700 (28)
	35-44	663 (27)
	45-54	363 (15)
	55-64	197 (8)
	65+	50 (2)
**Education level (N=4329)**		
	No high school	20 (0.5)
	High school	391 (9)
	Associates degree, Technikon, or Technical	233 (5.4)
	University (Bachelor)	1508 (35)
	University (Master)	1372 (32)
	Graduate degree (MD or PhD)	639 (15)
	Other	166 (3.8)
**Type of organization (N=4329)**		
	Academic	505 (12)
	Advocacy group	37 (0.85)
	International nongovernmental organization (iNGO)	146 (3.4)
	Marketing, Media, or Creative	146 (3.4)
	Ministry of Health, or Government	447 (10)
	Local nongovernmental organization (NGO)	266 (6.1)
	Private sector	600 (14)
	Self-employed	400 (9)
	Student	427 (9.9)
	UN Organization (ie, UNICEF^a^ and WHO^b^)	122 (2.82)
	Unemployed	328 (7.6)
	Other or not applicable^c^	905 (21)
**Countries represented (top 10; N=4329)**		
	United States	986 (22)
	Chile	208 (4.8)
	India	196 (4.5)
	Canada	153 (3.5)
	Nigeria	140 (3.2)
	United Kingdom	111 (2.6)
	Brazil	104 (2.4)
	Kenya	102 (2.4)
	Ghana	85 (2)
	Egypt	81 (1.9)
**UNICEF regions^d^ (N=4311)**		
	East Asia and Pacific	414 (9.6)
	Eastern Europe and Central Asia	232 (5.4)
	Western Europe	582 (14)
	Latin American and Caribbean	597 (14)
	Middle East and North Africa	321 (7.5)
	North America	1121 (26)
	South Asia	321 (7.5)
	Eastern and Southern Africa	398 (9.2)
	West and Central Africa	325 (0.54)

^a^UNICEF: United Nations International Children’s Emergency Fund.

^b^WHO: World Health Organization.

^c^Includes organizations that did not fit predetermined classifications or were not identifiable.

^d^UNICEF regional classifications [17].

### KPIs for Learners at Baseline

**Figure 4:**
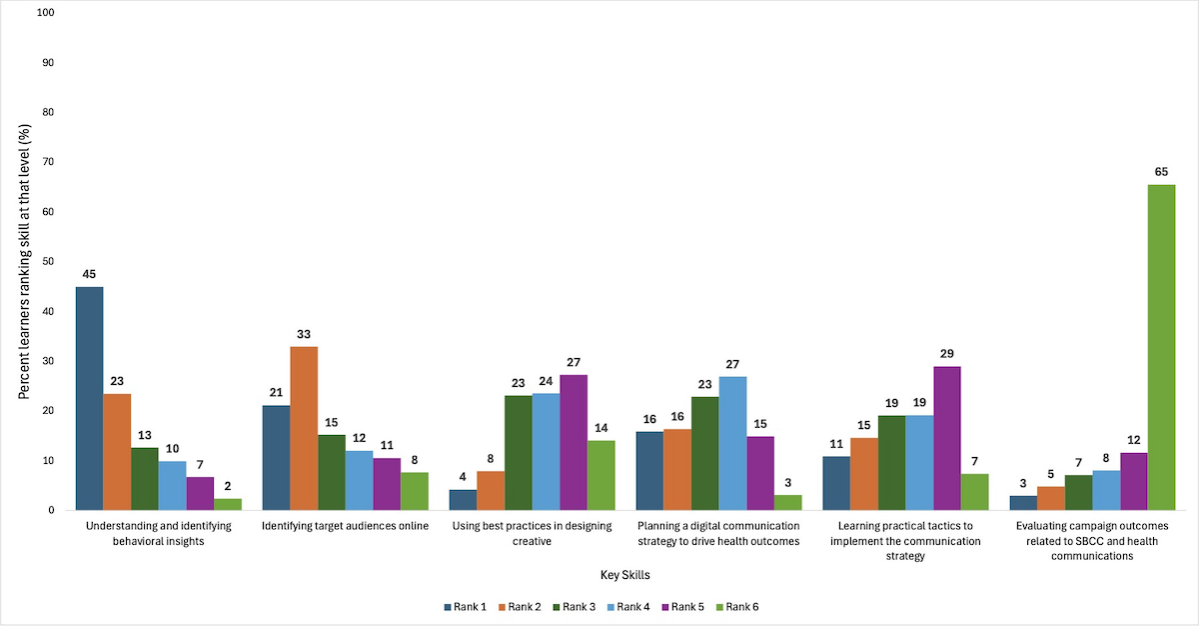
Skills ranked “most important to learn” by Digital Media for Health Outcomes course learners before taking the course (baseline).

When asked to rank which skills they wanted to learn from the course, learners ranked “understanding and identifying behavioral insights” as the most important skill to learn (Figure 4). Learners ranked “identifying target audiences online” second, “using best practices in designing creative [campaign images and graphics]” and “planning a digital communication strategy to drive health outcomes” third and fourth, “learning practical tactics to implement the communication strategy” fifth, and “evaluating campaign outcomes related to SBCC and health communications” last. Overall, most learners at baseline were confident in using these skills and found using digital media to drive health outcomes to be important, easy, and effective, and reported they were likely to do so (Table 3).

**Table 3:** Percent point change of learners reporting high confidence in skills before and after taking the Digital Media for Health Outcomes course (April 2025).

Questions			Baseline confidence, n (%)				Endline confidence, n (%)				Percent point change in high confidence (95% CI)		*P* value
			Low (0-5)		High (6-10)		Low (0-5)		High (6-10)				
**Please rate how confident you are in each of the following skills on a scale of 0-10**													
	Understanding and identifying behavioral insights	846 (20)		3483 (80)		23 (3.6)		611 (96)		16% (14%-18%)		<.001	
	Identifying a target audience online	960 (22)		3369 (78)		20 (3.2)		614 (97)		19% (17%-21%)		<.001	
	Planning a digital communication strategy to drive health outcomes	1494 (35)		2835 (66)		23 (3.4)		611 (96)		31% (29%-33%)		<.001	
	Using practical tactics to implement the communication strategy	1428 (33)		2901 (67)		21 (3.3)		613 (97)		30% (28%-32%)		<.001	
	Evaluating campaign outcomes related to SBCC^a^/health communications	1334 (31)		2995 (69)		21 (3.3)		613 (97)		28% (26%-29%)		<.001	
**On a scale of 0-10**													
	How confident are you in your ability to use digital media to drive health outcomes?	867 (20)		3462 (80)		11 (1.7)		623 (98)		18% (17%-20%)		<.001	
	How important do you think it is for your organization to use digital media to drive health outcomes?	241 (5.6)		4088 (94)		6 (0.95)		628 (99)		5% (4%-6%)		<.001	
	How easy is it in your workplace to integrate digital media as a tool to drive health outcomes?	535 (12)		3794 (88)		15 (2.4)		619 (97)		9% (7%-11%)		<.001	
	How likely are you to use digital media to reach your organization’s health goals?	321 (7.4)		4008 (93)		8 (1)		626 (99)		6% (5%-7%)		<.001	
	How effective is digital media in helping you reach your organization’s health goals?	410 (9.5)		3919 (91)		8 (1)		626 (99)		8% (7%-9%)		<.001	

^a^SBCC: social and behavioral change communication.

When asked how often they used digital media to drive health outcomes, 39% (1668/4329) learners responded “never or rarely,” 34% (1467/4329) responded “sometimes,” and 28% (1194/4329) responded “often or always.” Stratifying by learners’ organization type, we saw that students were significantly less likely to use digital media to drive health outcomes. Similarly, when asked how often they evaluated health outcomes in response to digital campaigns, 61% (2551/4157) responded “never or rarely,” 28% (1156/4157) responded “sometimes,” and 11% (450/4157) responded “often or always” (Table 4). Stratification by the learner organization did not show any significant differences.

**Table 4:** Frequency of digital media use and evaluation among learners before and after taking the Digital Media for Health Outcomes course (April 2025).

Questions			Baseline, n (%)		Endline, n (%)		Percent point change between baseline and endline (95% CI)		*P* value
**How often do you use digital media to drive health outcomes?^a^**									
	Never or rarely	1668 (39)		206 (33)		–6% (–10% to –2%)		.004	
	Sometimes	1467 (34)		217 (34)		3% (–4% to 4%)		.89	
	Often or always	1194 (28)		211 (33)		6% (2% to 10%)		.003	
**How often do you evaluate health outcomes in response to digital campaigns?^b^**									
	Never or rarely	2551 (61)		293 (49)		–12% (–16% to –8%)		<.001	
	Sometimes	1156 (28)		204 (34)		7% (2% to 11%)		.001	
	Often or always	450 (11)		97 (16)		6% (2% to 9%)		<.001	

^a^Baseline N=4329, endline n=634.

^b^Baseline n=4157, endline n=594.

### Demographics of Course Completers

**Figure 5:**
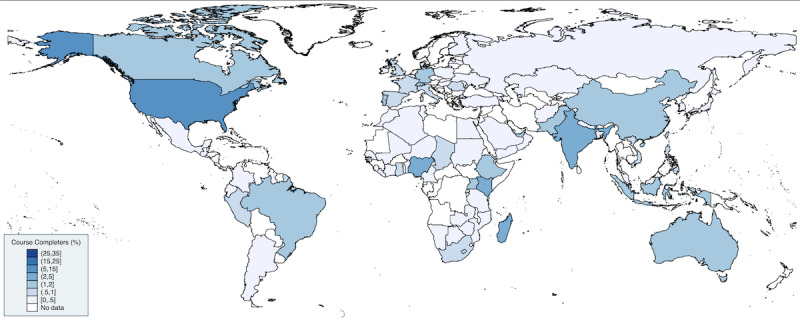
Countries represented by Digital Media for Health Outcomes course completers at endline, April 2025 (n=634).

Of the 682 (93%) learners who started the endline survey, 634 learners completed it as of April 2025 (Table 5). The English (316/634, 50%) and Spanish (234/634, 37%) versions of the course had the most completers. Most completers identified as women (464/634, 73%), were between 18 and 34 years of age (379/548, 69%), and had earned at least their bachelor’s degrees (399/634, 63%). Many course completers were students (226/634, 36%) or worked for ministries of health and government agencies (30/634, 4.7%), academic organizations (59/634, 9%), or the private sector (56/634, 9%). The top three countries represented were Chile (240/634, 38%), the United States (69/634, 11%), and Madagascar (26/634, 4%), and the top 3 regions were Latin America and Caribbean (271/634, 43%), North America (79/634, 13%), and Eastern and Southern Africa (76/634, 12%; Figure 5).

**Table 5:** Learner demographics for the Digital Media for Health Outcomes course at endline (April 2025).

Demographic			Baseline survey results, n (%)
**Survey language (n=634)**			
	Arabic	6 (0.95)	
	Bahasa Indonesian	6 (0.95)	
	Chinese	7 (1.1)	
	English	316 (50)	
	French	49 (7.7)	
	Portuguese	10 (1.6)	
	Russian	6 (0.95)	
	Spanish	234 (37)	
	Swahili	0 (0)	
**Gender (n=634)**			
	Men	156 (25)	
	Women	464 (73)	
	Other (nonbinary, self-describing, prefer not to say)	14 (2.2)	
**Age (y; n=548)**			
	13-17	4 (0.73)	
	18-24	265 (48)	
	25-34	114 (21)	
	35-44	103 (19)	
	45-54	42 (7.7)	
	55-64	17 (3.1)	
	65+	3 (0.55)	
**Education level (n=634)**			
	No high school	0 (0)	
	High school	166 (26)	
	Associates degree, Technikon, or Technical	31 (4.9)	
	University (Bachelor)	203 (32)	
	University (Master)	153 (24)	
	Graduate degree (MD or PhD)	46 (7.3)	
	Other	35 (5.5)	
**Type of organization (n=634)**			
	Academic	59 (9.3)	
	Advocacy group	2 (0.32)	
	International nongovernmental organization (iNGO)	30 (4.7)	
	Marketing, Media, or Creative	30 (4.7)	
	Ministry of Health or Government	30 (4.7)	
	Local nongovernmental organization (NGO)	32 (5.1)	
	Private sector	56 (8.8)	
	Self-employed	24 (3.8)	
	Student	226 (36)	
	UN Organization (ie, UNICEF^a^ and WHO^b^)	14 (2.2)	
	Unemployed	37 (5.8)	
	Other or not applicable	52 (8.2)	
**Countries represented (top 10; n=634)**			
	Chile	240 (38)	
	United States	69 (11)	
	Madagascar	26 (4.1)	
	India	19 (3)	
	Kenya	16 (2.5)	
	Nigeria	13 (2.1)	
	Uganda	12 (1.9)	
	Indonesia	11 (1.7)	
	Canada	10 (1.6)	
	Ethiopia	10 (1.6)	
**UNICEF regions represented^c^ (n=631)**			
	East Asia and Pacific	47 (7.5)	
	Eastern Europe and Central Asia	16 (2.5)	
	Western Europe	57 (9)	
	Latin American and Caribbean	271 (43)	
	Middle Easte and North Africa	17 (2.7)	
	North America	79 (13)	
	South Asia	32 (5.1)	
	Eastern and Southern Africa	76 (12)	
	West and Central Africa	36 (5.7)	

^a^UNICEF: United Nations International Children’s Emergency Fund.

^b^WHO: World Health Organization.

^c^UNICEF regional classifications [17].

### KPIs for Course Completers

There was a significant increase in skill confidence among course completers compared with baseline learners (Table 3). Respondents at endline were significantly more confident in all skills, especially planning a digital communication strategy, with 66% (2835/4329) reporting high confidence at baseline compared to 96% (611/634) at endline (+16%, 95% CI 14%-18%, *P*<.001), using practical tactics to implement that strategy (+30%, 95% CI 19%-33%, *P*<.001), and evaluating health outcomes after campaign implementation (+28%, 95% CI 26%-29%, *P*<.001). Other KPIs also showed significant improvement from baseline to endline. Learners were significantly more confident in their ability to use digital media (+18%, 95% CI 17%-20%, *P*<.001). Similarly, more endline respondents indicated using digital media was important (+5%, 95% CI 4%-6%, *P*<.001), easy (+9%, 95% CI 7%-11%, *P*<.001), effective (+8%, 95% CI 7%-9%, *P*<.001), and that they were likely to use it (+6%, 95% CI 5%-7%, *P*<.001).

When asked how often they used digital media to drive health outcomes, 33% (206/634) responded “never or rarely,” 34% (217/634) responded “sometimes,” and 33% (211/634) responded “often or always.” (Table 4). Stratifying by learners’ organization type showed that students were significantly less likely to use digital media as compared with other learners. When asked how often they evaluated health outcomes in response to digital campaigns, 50% (293/594) responded “never or rarely,” 34% (204/594) responded “sometimes,” and 16% (97/594) responded “often or always.” Stratification by organization type did not reveal any differences in the frequency of evaluation. From baseline to endline, there was a significant decrease in the number of respondents who never or rarely used digital media (–6%, *P=*.004), and a significant increase in the number of respondents who often or always used digital media (+6%, *P=*.003; Table 4). Similarly, there was a significant decrease in the number of respondents who never or rarely evaluated health outcomes from baseline to endline (–12%, *P*<.001), and significant increases among respondents who sometimes (+7%, *P=*.001) or often or always evaluated outcomes (+6%, *P*<.001).

### Longitudinal Analysis of Course Completers

We compared baseline and endline survey responses for 360 (of 634) completers for whom we could link unique survey submissions (Table 6). Of these, most completed the English (210/360, 58%) and Spanish (120/360, 33%) versions of the course, 79% (285/360) identified as women, 55% (149/271) were between 18 and 24 years old, and most had completed at least their bachelor’s degree (252/360, 70%). The top-represented country was Chile (124/360, 34%), followed by the United States (43/360, 12%).

**Table 6:** Learner demographics matched between baseline and endline surveys for the Digital Media for Health Outcomes course (April 2025).

Demographic		Survey results, n (%)
**Survey language (n=360)**		
	Arabic	1 (0.28)
	Bahasa Indonesian	3 (0.83)
	Chinese	2 (0.56)
	English	210 (58)
	French	18 (5)
	Portuguese	1 (0.28)
	Russian	5 (1.4)
	Spanish	120 (33)
	Swahili	0 (0)
**Gender (n=360)**		
	Men	68 (19)
	Women	285 (79)
	Other (nonbinary, self-describing, prefer not to say)	7 (1.9)
**Age (y; n=271)**		
	13-17	2 (0.74)
	18-24	149 (55)
	25-34	40 (15)
	35-44	59 (22)
	45-54	16 (5.9)
	55-64	4 (1.5)
	65+	1 (0.37)
**Education level (n=360)**		
	No high school	0 (0)
	High school	87 (24)
	Associates degree, Technikon, or Technical	23 (6.4)
	University (Bachelor)	119 (33)
	University (Master)	92 (26)
	Graduate degree (MD or PhD)	16 (4.4)
	Other	23 (6.4)
**Type of organization (n=360)**		
	Academic	33 (9.2)
	Advocacy group	0 (0)
	International nongovernmental organization (iNGO)	20 (5.6)
	Marketing, Media, or Creative	3 (0.83)
	Ministry of Health or Government	26 (7.2)
	Local nongovernmental organization (NGO)	13 (3.6)
	Private sector	33 (9.2)
	Self-employed	19 (5.3)
	Student	117 (33)
	UN Organization (ie, UNICEF^a^ and WHO^b^)	22 (6.1)
	Unemployed	22 (6.1)
	Other or not applicable^c^	52 (14)
**Countries represented (top 10; n=360)**		
	Chile	124 (34)
	United States	43 (12)
	Madagascar	15 (4.2)
	Kenya	13 (3.6)
	India	11 (3.1)
	Philippines	10 (2.8)
	Canada	9 (2.5)
	Nigeria	9 (2.5)
	Pakistan	9 (2.5)
	Indonesia	8 (2.2)
**UNICEF regions^d^ (n=359)**		
	East Asia and Pacific	34 (9.5)
	Eastern Europe and Central Asia	10 (2.8)
	Western Europe	27 (7.5)
	Latin American and Caribbean	137 (38)
	Middle East and North Africa	9 (2.5)
	North America	52 (15)
	South Asia	26 (7.2)
	Eastern and Southern Africa	47 (13)
	West and Central Africa	17 (4.7)

^a^UNICEF: United Nations International Children’s Emergency Fund.

^b^WHO: World Health Organization.

^c^Includes organizations that did not fit predetermined classifications or were not identifiable.

^d^UNICEF regional classifications [17].

As in the main analysis, course completers reported significantly higher confidence in key skills compared to baseline (Table 7). Notably, completers saw increases in confidence of 14% percentage points for planning a digital communications strategy (95% CI 0.09-0.19, *P*<.001) and 16% percentage points for outcome evaluation (95% CI 0.1-0.21, *P*<.001). Similarly, completers’ confidence in their ability to use digital media, the likelihood they would do so, and their perception of its effectiveness improved significantly after taking the course (Table 7). Between baseline and endline, no significant change was seen in how often course completers used digital media for health campaigns or evaluated health outcomes following those campaigns.

**Table 7:** Percent point change of learners reporting high confidence for direct comparison of the Digital Media for Health Outcomes course completers, baseline to endline.

Questions		Baseline confidence, n (%)			Endline confidence, n (%)			Percent point change in high confidence (95% CI)		*P* value
		Low (0-5)	High (6-10)	Low (0-5)		High (6-10)				
**Please rate how confident you are in each of the following skills on a scale of 0-10**										
	Understanding and identifying behavioral insights	58 (16)	302 (84)	30 (8.3)		330 (92)	8% (3% to 13%)		.001	
	Identifying a target audience online	52 (14)	308 (86)	6 (1.7)		354 (98)	13% (9% to 17%)		<.001	
	Planning a digital communication strategy to drive health outcomes	78 (22)	282 (78)	27 (7.5)		333 (93)	14% (9% to 19%)		<.001	
	Using practical tactics to implement the communication strategy	75 (21)	285 (79)	29 (8.1)		331 (92)	13 (8% to 18%)		<.001	
	Evaluating campaign outcomes related to SBCC^a^/health communications	83 (23)	277 (77)	27 (7.5)		333 (93)	16% (10% to 21%)		<.001	
**On a scale of 0-10**										
	How confident are you in your ability to use digital media to drive health outcomes?	43 (12)	317 (88)	21 (5.8)		339 (94)	6% (2% to 10%)		.004	
	How important do you think it is for your organization to use digital media to drive health outcomes?	11 (3.1)	349 (97)	20 (5.6)		340 (94)	–2.5% (–55% to 0.5%)		.10	
	How easy is it in your workplace to integrate digital media as a tool to drive health outcomes?	30 (8.3)	830 (92)	25 (6.9)		335 (93)	14% (–2% to 0.5%)		.49	
	How likely are you to use digital media to reach your organization’s health goals?	14 (4.2)	345 (96)	2 (0.56)		358 (99)	3.6% (1.4% to 5.8%)		.001	
	How effective is digital media in helping you reach your organization’s health goals?	22 (6.1)	338 (94)	23 (6.4)		337 (94)	54% (48% to 59%)		<.001	

^a^SBCC: social and behavioral change communication.

### Course Evaluation

Course completers were asked to evaluate the overall course and specific modules. Generally, it took most learners less than 1 month to complete the course (348/634, 55%) and <4 hours to complete each module (475/634, 75%). Most respondents (570/634, 90%) reported using a laptop or computer to take the course, and 90% (570/634) of learners agreed that, from where they took the course, the content was easy to load and view.

Over 70% (444/634) of respondents reported that they had applied some or all skills from the course to their work. Commonly applied skills included “using behavioral insights to develop social and behavior change communication” (190/634, 30% and 133/634, 21% indicated often or always, respectively), “developing a strategic brief” (197/634, 31% and 139/634, 22%), and “using Canva or a similar platform to create campaign assets” (184/634, 29% and 228/634, 36%).

**Figure 6:**
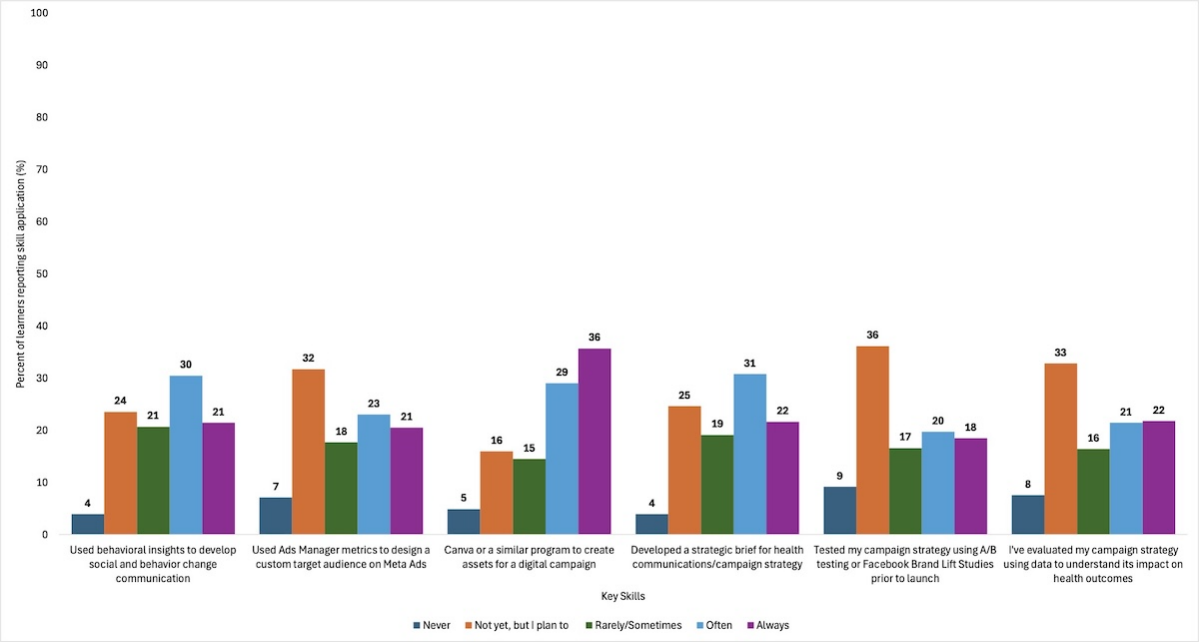
Skills applied by course completers since taking the Digital Media for Health Outcomes course, as of April 2025.

When asked if they had used Meta to design custom target audiences, used A/B testing to assess their campaign strategy, or evaluated health outcomes following their campaign, the most common response was “not yet, but I plan to” (197/634, 31%, 228/634, 36%, and 208/634, 33% respectively; Figure 6).

Overall, 92% (583/634) of course completers felt that the course had met most, all, or exceeded their expectations. When asked how satisfied they were with individual course elements, most learners agreed they were satisfied with the instructor videos (577/634, 91%), case studies (583/634, 92%), the Coursera interface (570/634, 90%), course pacing (577/634, 91%), homework assignments (558/634, 88%), quizzes (552/634, 87%), and translation (539/634, 85%). The peer-review process was the least satisfactory, with only 78% (494/634) satisfaction. Anonymous (and optional) comments at the end of the survey cited wishes that all assignments could be reviewed by course instructors or content experts and graded at more regular intervals rather than by the Coursera peer-review system. Individual modules were also well-received by respondents. For each module, the majority of course completers agreed that the material was new to them, easy to understand, and relevant to their work (Table S1 in Multimedia Appendix 2).

### Course Recommendation

When asked how likely they were to recommend the course to a colleague or friend, 85% (539/634) indicated that they were likely to do so. When asked whether they would share their certificate of completion (if applicable) on LinkedIn, 64% (406/634) indicated that they would (159/634, 25% indicated “not applicable” either because they chose to complete the course without certification or because they do not have a LinkedIn page).

## Discussion

### Principal Findings

The DMHO course was designed to be accessible and useful to public health practitioners with limited experience in behavioral evidence-based health communication. Notably, the course has reached thousands of learners from countries in Asia, Africa, and Latin America. A course that is available online, free of charge, and in multiple languages has the potential to make useful training available to audiences worldwide.

The COVID-19 pandemic demonstrated the need for strategic campaigns on digital media, specifically social media, to reach diverse audiences with essential information, especially in the context of widespread online misinformation. Organizations must be equipped both to communicate quickly and effectively during a crisis and to maintain credibility as a trusted voice for public health information. The DMHO course provides an evidence-based framework for the development of campaigns that can be applied to systematically target health behaviors, offer compelling information, and promote demand for services. Encouragingly, the evaluation revealed that the course improved self-reported confidence among learners from baseline to endline, along with self-reported endorsement that using digital media to drive health outcomes was important, easy, and effective. Direct comparison of baseline versus endline survey results for learners who completed both revealed similarly promising results to overall analyses. Learners’ perceived ability to apply key skills improved from baseline to endline, as did their confidence in using digital media, how effective they believed it to be, and how likely they were to use it. Moreover, more respondents indicated they were likely to use digital media to achieve their organization’s goals at endline than at baseline. These results are especially promising in the context of our diverse learner pool, about half of whom are based in Africa, Latin America, and the Caribbean.

Many organizations, including our partners on the DMHO course, have used digital campaigns to share pertinent information about health topics and encourage recommended behaviors. While few academic studies have documented the direct impact of such campaigns on health outcomes, targeted social and behavior change communication has been used to improve health knowledge, attitudes, and health-seeking behaviors [9,18-20]. To develop the DMHO course, we combined evidence from multiple experts and experiences to produce a practice-based training that walks learners through the communication process, emphasizing behavioral theory and encouraging empirical evaluation of health outcomes. Designing and implementing campaigns without an evidence-based framework can limit their impact or have unintended consequences [21,22]. Skills-based massive open online courses (MOOCs), like DMHO, have the capacity to improve skills and shift attitudes toward important topics within and beyond public health. Similar evaluations have described successful launches of MOOCs to improve skill-based knowledge and reach a wide and diverse audience at a scale not possible with in-person training [23-25]. These evaluations noted that MOOC success is predicated upon engaging learners with creative and interactive teaching styles, and ensuring material is accessible in terms of format and language, all factors that we considered and tried to address when developing this course.

Our results highlight the need for further capacity strengthening, particularly in terms of applying best practices and evaluating impact. Most practitioners believe these skills are important, but many do not apply them in practice. While course completers reported they were more likely to use digital media often or always than baseline learners, most reported rarely doing so. Notably, many of the learners who completed the course were university students from Chile, and while they may not be using digital media to drive health outcomes now, the course may have exposed them to skills they could apply in the future. Similarly, many course completers indicated they were sometimes, often, or always evaluated health outcomes; however, nearly half reported never or rarely doing so. Evaluation of health outcomes is an important step in the development and implementation of effective digital health campaigns, but it can be challenging, particularly when the intended outcome is a behavior change. More information is needed to understand the say-do gap in the implementation of digital health communications among public health agencies, and how we can encourage public health practitioners to evaluate the impact of their campaigns.

This study has several limitations. All data are self-reported and subject to social desirability and bias, representing those who register and enroll for such a course. The diverse baseline sample mitigates this limitation, as do the results of the completer subanalysis. The subanalysis is limited by its small sample size, which is a function of the anonymity of the surveys. The global and likely heterogeneous nature of our results may obscure regional differences at baseline and endline assessments. While regional analysis was beyond the scope of this initial performance evaluation, we anticipate conducting future analyses at the regional level as course completion increases across regions.

Furthermore, our content is insufficient to keep pace with the dynamic and changing environment of social media platforms and the different ways that various platforms leverage their audience through advertising and other algorithms. Nonetheless, the course provides a foundation for how to approach any SBCC campaign through whatever digital means are prevalent for a target audience. Further investigation is also needed to understand why there is such a high level of enrollment yet a relatively small percentage of completers. Data provided by Coursera course management dashboards shows that most learners who do not complete the course drop off in the first module, either immediately after registering for the course (before the baseline survey) or before the first homework assignment. While this is consistent with other Coursera courses presented by Yale University, a deeper analysis is warranted to promote sustained enrollment in the course through completion.

Finally, this performance evaluation is not sufficient to assess the “practice gap.” Understanding the long-term outcomes of digital campaign development training is essential to improving communication capacity among public health organizations and should be the focus of future studies.

### Conclusion

The DMHO course suggests the potential of a practical, theory-based massive online course to improve public health practitioners’ confidence and skills in using digital media to drive health outcomes. However, the need for further capacity strengthening is clear. Digital health communication is an increasingly useful tool for public health practitioners, one which—if wielded strategically—has the continued ability to reach different audiences with the critical information they need and improve health outcomes.

## Appendix

Multimedia Appendix 1 Questions for baseline and endline surveys instruments.

Multimedia Appendix 2 Table S1. Module-specific evaluation.

## Abbreviations

CDC: Centers for Disease Control and Prevention
DMHO: Digital Media for Health Outcomes
SBCC: social and behavioral change communication
UNICEF: United Nations’ International Children’s Emergency Fund
WHO: World Health Organization

## Data Availability

Deidentified aggregated data will be available for download from Open Science Framework [26].

## References

[1] Ferreira Caceres MM, Sosa JP, Lawrence JA, Sestacovschi C, Tidd-Johnson A, Rasool MHU, Gadamidi Vinay Kumar, Ozair Saleha, Pandav Krunal, Cuevas-Lou Claudia, Parrish Matthew, Rodriguez Ivan, Fernandez Javier Perez (2022). The impact of misinformation on the COVID-19 pandemic. AIMS Public Health.

[2] (2020). Managing the COVID-19 infodemic: promoting healthy behaviours and mitigating the harm from misinformation and disinformation. World Health Organization.

[3] (2025). SEO.AI.

[4] (2025). Most popular social networks worldwide as of February 2025, by number of monthly active users (in millions). Statista.

[5] Global social media statistics. DataReportal.

[6] Tsao S, Chen H, Tisseverasinghe T, Yang Y, Li L, Butt ZA (2021). What social media told us in the time of COVID-19: a scoping review. Lancet Digit Health.

[7] González-Padilla Daniel A, Tortolero-Blanco L (2020). Social media influence in the COVID-19 pandemic. Int Braz J Urol.

[8] Ho HY, Chen YL, Yen CF (2020). Different impacts of COVID-19-related information sources on public worry: an online survey through social media. Internet Interv.

[9] Winters M, Christie S, Lepage C, Malik AA, Bokemper S, Abeyesekera S, Boye Brian, Moini Midhat, Jamil Zara, Tariq Taha, Beresh Tamara, Kazymyrova Ganna, Palamar Liudmyla, Paintsil Elliott, Faller Alexandra, Seusan Andreea, Bonnevie Erika, Smyser Joe, Khan Kadeem, Gulaid Mohamed, Francis Sarah, Warren Joshua L, Thomson Angus, Omer Saad B (2023). Persuasive COVID-19 vaccination campaigns on Facebook and nationwide vaccination coverage in Ukraine, India, and Pakistan. PLOS Glob Public Health.

[10] Malik AA, Ahmed N, Shafiq M, Elharake JA, James E, Nyhan K, Paintsil Elliott, Melchinger Hannah Camille, Team Yale Behavioral Interventions, Malik Fauzia A, Omer Saad B (2023). Behavioral interventions for vaccination uptake: a systematic review and meta-analysis. Health Policy.

[11] Rosa WE, Levoy K, Doyon K, McDarby M, Ferrell BR, Parker PA, Sanders Justin J, Epstein Andrew S, Sullivan Donald R, Rosenberg Abby R (2023). Integrating evidence-based communication principles into routine cancer care. Support Care Cancer.

[12] Roundtable on Population Health Improvement, Board on Population Health and Public Health Practice, Institute of Medicine (2015). Effective messaging strategies: a review of the evidence. Communicating to Advance the Public's Health: Workshop Summary.

[13] Riera R, de Oliveira Cruz Latorraca C, Padovez RCM, Pacheco RL, Romão Davi Mamblona Marques, Barreto JOM, Machado Maria Lúcia Teixeira, Gomes Romeu, da Silva Silvio Fernandes, Martimbianco Ana Luiza Cabrera (2023). Strategies for communicating scientific evidence on healthcare to managers and the population: a scoping review. Health Res Policy Syst.

[14] Winters M, Christie S, Melchinger H, Iddrisu I, Al Hassan H, Ewart E, Mosley Lateefah, Alhassan Rabiu, Shani Ndeeya, Nyamuame Dela, Lepage Chelsey, Thomson Angus, Atif Anastasiia Nurzhynska, Omer Saad B (2025). Debunking COVID-19 vaccine misinformation with an audio drama in Ghana, a randomized control trial. Sci Rep.

[15] Coursera.

[16] Qualtrics.

[17] (2023). Regional classifications. UNICEF.

[18] Winters M, Christie S, Melchinger H, Arias N, Lirman L, Thomson A, Omer Saad B (2024). Moral foundations messaging to improve vaccine attitudes: an online randomized experiment from Argentina. PLOS Glob Public Health.

[19] Winters M, Sochoń-Latuszek A, Nurzhynska A, Yoruk K, Kukuła K, Bahruddinov M, Kusek Aleksandra, Kleszczewska Dorota, Dzielska Anna, Maciejewski Tomasz, Mazur Joanna, Melchinger Hannah, Kinsman John, Kramarz Piotr, Christie Sarah, Omer Saad B (2024). "Vaccinating your child during an emergency is more important than ever": a randomised controlled trial on message framing among Ukrainian refugees in Poland, 2023. Euro Surveill.

[20] Hansen RK, Baiju N, Gabarron E (2023). Social media as an effective provider of quality-assured and accurate information to increase vaccine rates: systematic review. J Med Internet Res.

[21] Swire-Thompson B, Miklaucic N, Wihbey JP, Lazer D, DeGutis J (2022). The backfire effect after correcting misinformation is strongly associated with reliability. J Exp Psychol Gen.

[22] Ruggeri K, Vanderslott S, Yamada Y, Argyris YA, Većkalov B, Boggio PS, Fallah Mosoka P, Stock Friederike, Hertwig Ralph (2024). Behavioural interventions to reduce vaccine hesitancy driven by misinformation on social media. BMJ.

[23] Ferrari E, Ballegeer AM, Corrochano D, Fuertes MÁ, Herrero Teijón P, Delgado Martín ML, Sánchez SA (2024). Improvement of attitudes and skills using a MOOC about the basic science of climate change. Humanit Soc Sci Commun.

[24] Herbert M, Smith IM, Guest C (2025). Transforming improvement training at scale with essential digital training skills. BMJ Open Qual.

[25] Huang H, Qi D (2025). Is MOOC really effective? Exploring the outcomes of MOOC adoption and its influencing factors in a higher educational institution in China. PLoS One.

[26] Digital media for health outcomes: evaluation study of a massive online open course. Open Science Framework.

