# Supplementary material for: Digital Media for Health Outcomes: Evaluation Study of a Massive Online Open Course
Source: J Med Internet Res. 2026 Jun 25;28:e85016. doi: 10.2196/85016 (PMC13351640; doi:10.2196/85016)
Supplement: Multimedia Appendix 1 [file jmir_v28i1e85016_app1.pdf]

DMHO Baseline Survey (Public)

Start of Block: Default Question Block

Q1 Welcome to the Yale Digital Media for Health Outcomes course. Before the course begins, we would like to ask you some questions about your experience using social and behavior change communications (SBCC) in digital media. By digital media, we mean any communication that can be created, viewed, and distributed on a digital electronics communication device, such as a computer or mobile phone. These digital communications can be featured on various social media platforms, such as Facebook, Twitter, or TikTok.   We will use the answers to this survey to study the impact of the course over time. Your answers to these questions will be kept confidential. Thank you for your participation!

Page Break

End of Block: Default Question Block

Start of Block: Personal Information

Q2 What is your gender identity?

- ○ Male (1)
- ○ Female (2)
- ○ Non-binary (3)
- ○ I prefer to self-describe: (4) ____
- ○ Prefer not to say (5)

Page Break

Q3 What is your age (in years)?

- 13-17 (4)
- 18-24 (5)
- 25-34 (6)
- 35-44 (7)
- 45-54 (8)
- 55-64 (9)
- 65+ (10)

Page Break

Q4 What is your highest level of education?

- ◯ No high school (1)
- ◯ High school (2)
- ◯ Associates degree/Technikon/Technical (3)
- ◯ University: Bachelor (4)
- ◯ University: Master (5)
- ◯ Graduate degree: MD, PhD (6)
- ◯ Other: (7) ____

End of Block: Personal Information

Start of Block: Contact Information

*

Q25 What is your preferred email address for follow-up? (This question is optional)

____

End of Block: Contact Information

Start of Block: Job Information

Q5 What is the name of the organization you currently work for?

____

Page Break

Q36 What kind of organization do you work for? (Choose the most applicable classification)

- ○ Academic (3)
- ○ Research Organization (17)
- ○ Non-governmental organization (NGO) (1)
- ○ International NGO (i.e. Save the Children, CARE) (2)
- ○ UN Organization (i.e. UNICEF, UNHCR) (14)
- ○ Ministry of Health/Government (4)
- ○ Private sector (5)
- ○ Marketing/Media/Creative Group (11)
- ○ Advocacy group (15)
- ○ Student (9)
- ○ Self-employed (8)
- ○ Unemployed (10)
- ○ Other: (13) ____

Page Break

Q6 What is your role/job title?

____

Page Break

Q24 What country are you currently based in?

▼ Afghanistan (1) ... Zimbabwe (1357)

Page Break

Q29 How many years of work experience do you have in your current field?

____

Page Break

Q20 How many years of work experience do you have with Social and Behavior Change Communication (SBCC) using digital/social media?   SBCC can include public health campaigns, health communications, information and educational communication (IEC) material, demand generation, communication for development (C4D), or any community engagement/outreach to drive health outcomes.

◯ I have no prior experience with SBCC in digital media (4)

◯ Number of years: (5) ____

Page Break

Q28 Which, if any, digital/social media platforms have you used for health-related social behavior change communications? Please select as many as apply.

- ☐ Facebook (1)
- ☐ Instagram (2)
- ☐ TikTok (3)
- ☐ Twitter (4)
- ☐ YouTube (5)
- ☐ LinkedIn (6)
- ☐ SnapChat (7)
- ☐ Other (please specify): (8) ____

End of Block: Job Information

Start of Block: Course questions

Q8 How did you hear about this course?

- ○ Yale Institute for Global Health (1)
- ○ Meta (2)
- ○ LinkedIn, or other social media platform (3)
- ○ Friend or colleague recommended (4)
- ○ Manager at work recommended/required (5)
- ○ PSI (6)
- ○ UNICEF (7)
- ○ Africa CDC (8)
- ○ WHO/PAHO (9)
- ○ Ad Council (10)
- ○ Local NGO (11)
- ○ Government ministry/agency (12)
- ○ Coursera (14)
- ○ Course alumni recommended (15)
- ○ Other (please specify): (13) ____

Page Break

Q11 For each of the following skills, please rate how confident you are on a scale of 0-10:

	0 (1)	1 (2)	2 (3)	3 (4)	4 (5)	5: Neutral (6)	6 (7)	7 (8)	8 (9)	9 (10)	10 (11)
Understanding and identifying behavioral insights (1)	○	○	○	○	○	○	○	○	○	○	○
Identifying a target audience online (2)	○	○	○	○	○	○	○	○	○	○	○
Planning a digital communication strategy to drive health outcomes (3)	○	○	○	○	○	○	○	○	○	○	○
Practical tactics to implement the communication strategy (4)	○	○	○	○	○	○	○	○	○	○	○
Evaluating campaign outcomes (5)	○	○	○	○	○	○	○	○	○	○	○

Page Break

Q11 Which skills do you think are most important to learn? Please rank below (by dragging):
_______ Understanding and identifying behavioral insights (1)
_______ Identifying a target audience online (2)
_______ Using best practices in designing creatives (3)
_______ Planning a digital communication strategy to drive health outcomes (4)
_______ Learning practical tactics to implement the communication strategy (5)
_______ Evaluating campaign outcomes related to SBCC/health communications (6)

End of Block: Course questions

Start of Block: Course Effectiveness

Q13 How often do you use digital media to drive health outcomes?

- ○ Never (1)
- ○ Rarely (2)
- ○ Sometimes (3)
- ○ Often (4)
- ○ Always (5)

Page Break

Q31 How often do you evaluate health outcomes in response to your digital health campaigns?

- ○ Never (1)
- ○ Rarely (2)
- ○ Sometimes (3)
- ○ Often (4)
- ○ Always (5)

Page Break

Q12 On a scale from 0-10 (where 0 is least and 10 is most),

	0 (1)	1 (2)	2 (3)	3 (4)	4 (5)	5 (6)	6 (7)	7 (8)	8 (9)	9 (10)	10 (11)
How confident are you in your ability to use digital media to drive health outcomes? (2)	○	○	○	○	○	○	○	○	○	○	○
How important do you think it is for your organization to use digital media to drive health outcomes? (4)	○	○	○	○	○	○	○	○	○	○	○
How easy is it in your workplace to integrate digital media as a tool to drive health outcomes? (5)	○	○	○	○	○	○	○	○	○	○	○
How likely are you to use digital media to reach your organization's health goals? (1)	○	○	○	○	○	○	○	○	○	○	○
How effective is digital media in helping you reach your organization's health goals? (3)	○	○	○	○	○	○	○	○	○	○	○

End of Block: Course Effectiveness

Start of Block: Funding/Resources

Q14 Does your organization allocate internal funding to SBCC activities using digital media?

○ Yes (1)

○ No (2)

○ I don't know (3)

Display this question:

If Does your organization allocate internal funding to SBCC activities using digital media? = Yes

Q21 Was this dedicated SBCC funding available prior to the COVID-19 pandemic?

○ Yes (1)

○ No (2)

○ I don't know (3)

Page Break

Q15 Of the health initiatives or projects you support, roughly what percent of budget is spent on digital media to drive your health outcomes?

○ % (1) ____

○ I don't know (2)

Page Break

Q17 How many proposals or funding requests do you think your organization has submitted in the last 6 months to support SBCC activities using digital media?

- ○ Number of proposals: (4) ____
- ○ I am not involved in funding requests (5)
- ○ Not applicable (6)

End of Block: Funding/Resources

Start of Block: File upload

Q19 **Optional.** Please upload an example of a) a recent social media post or b) a recent campaign to drive health outcomes that you have been involved with in your organization.

End of Block: File upload

DMHO Endline Survey (Public)

Start of Block: Intro text

Q1 Congratulations on completing the Yale Digital Media for Health Outcomes course! In order to improve the course for future learners, we would like to ask you a few questions.   Your responses help us to understand the impact of this course over time and will be kept confidential.   Thank you for taking the time to complete this survey.

End of Block: Intro text

Start of Block: Demographic Information

Q2 What is your gender identity?

- ○ Male (1)
- ○ Female (2)
- ○ Non-binary (3)
- ○ Prefer to self-describe: (4) ____
- ○ Prefer not to say (5)

Page Break

Q3 What is your age (in years)?

- 13-17 (4)
- 18-24 (5)
- 25-34 (6)
- 35-44 (7)
- 45-54 (8)
- 55-64 (9)
- 65+ (10)

Page Break

Q4 What is your highest level of education?

- ○ No high school (1)
- ○ High school (2)
- ○ Associates degree/Technikon/Technical (3)
- ○ University: Bachelor (4)
- ○ University: Master (5)
- ○ Graduate degree: MD, PhD (6)
- ○ Other: (7) ____

End of Block: Demographic Information

Start of Block: Current work and experience

Q5 What is the name of the organization you currently work for?

____

Page Break

Q52 What kind of organization do you work for? (Choose the most applicable classification)

- ○ Academic (3)
- ○ Research Organization (17)
- ○ Non-governmental organization (NGO) (1)
- ○ International NGO (i.e. Save the Children, CARE) (2)
- ○ UN Organization (i.e. UNICEF, UNHCR) (14)
- ○ Ministry of Health/Government (4)
- ○ Private sector (5)
- ○ Marketing/Media/Creative Group (11)
- ○ Advocacy group (15)
- ○ Student (9)
- ○ Self-employed (8)
- ○ Unemployed (10)
- ○ Other: (13) ____

Page Break

Q6 What is your role/job title?

____

Page Break

Q7 What country are you currently based in?

▼ Afghanistan (1) ... Zimbabwe (1357)

Page Break

Q8 How many years of work experience do you have with Social and Behavior Change Communication (SBCC) using digital/social media?   SBCC can include public health campaigns, health communications, information and educational communication (IEC) material, demand generation, communication for development (C4D), or any community engagement/outreach to drive health outcomes.

○ I have no prior experience with SBCC in digital media (4)

○ Number of years: (5) ____

Page Break

Q9 How often do you use digital media to drive health outcomes?

- ◯ Never (1)
- ◯ Rarely (2)
- ◯ Sometimes (3)
- ◯ Often (4)
- ◯ Always (5)

Page Break

Q10 How often do you evaluate health outcomes in response to your digital health campaigns?

- Never (1)
- Rarely (2)
- Sometimes (3)
- Often (4)
- Always (5)

End of Block: Current work and experience

Start of Block: SBCC Skills

Q11 For each of the following skills, please rate how confident you are on a scale of 0-10 since taking the course:

	0 (1)	1 (2)	2 (3)	3 (4)	4 (5)	5: Neutral (6)	6 (7)	7 (8)	8 (9)	9 (10)	10 (11)
Understanding and identifying behavioral insights (1)	○	○	○	○	○	○	○	○	○	○	○
Identifying a target audience online (2)	○	○	○	○	○	○	○	○	○	○	○
Planning a digital communication strategy to drive health outcomes (3)	○	○	○	○	○	○	○	○	○	○	○
Practical tactics to implement the communication strategy (4)	○	○	○	○	○	○	○	○	○	○	○
Evaluating campaign outcomes (5)	○	○	○	○	○	○	○	○	○	○	○

Page Break

Q12 On a scale from 0-10 (where 0 is least and 10 is the most),

	0 (1)	1 (2)	2 (3)	3 (4)	4 (5)	5: Neutral (6)	6 (7)	7 (8)	8 (9)	9 (10)	10 (11)
How confident are you in your abilities to use digital media to drive health outcomes? (1)	○	○	○	○	○	○	○	○	○	○	○
How important do you think it is for your organization to use digital media to drive health outcomes? (2)	○	○	○	○	○	○	○	○	○	○	○
How easy is it in your workplace to integrate digital media as a behavior change tool? (3)	○	○	○	○	○	○	○	○	○	○	○
How likely are you to use digital media for health outcomes to reach your organization's goals? (4)	○	○	○	○	○	○	○	○	○	○	○
How effective is digital media in helping you reach your organization's health goals? (5)	○	○	○	○	○	○	○	○	○	○	○

End of Block: SBCC Skills

Start of Block: Course Evaluation

Q13 To what extent did the course match your expectations?

- ◯ It did not meet my expectations at all (1)
- ◯ It met some of my expectations, but not all (2)
- ◯ It met most of my expectations (3)
- ◯ It met all of my expectations (4)
- ◯ It exceeded my expectations (5)

Display this question:

If To what extent did the course match your expectations? = It did not meet my expectations at all

Or To what extent did the course match your expectations? = It met some of my expectations, but not all

Q14 Can you let us know why the course did not meet your expectations?

____

Page Break

Q15 In Module 1 you learned about *Behavioral Insights as a Foundation.* Please indicate to what extent you agree with the following statements:

	Strongly disagree (1)	Disagree (6)	Neutral (2)	Agree (3)	Strongly agree (4)
The material was new to me (1)	○	○	○	○	○
The module was easy to understand (2)	○	○	○	○	○
The course material was relevant to my work (3)	○	○	○	○	○

Page Break

Q16 In Module 2 you learned about *Crafting your Communication Strategy.* Please indicate to what extent you agree with the following statements:

	Strongly disagree (1)	Disagree (2)	Neutral (3)	Agree (4)	Strongly agree (5)
The material was new to me (1)	○	○	○	○	○
The module was easy to understand (2)	○	○	○	○	○
The course material was relevant to my work (3)	○	○	○	○	○

Page Break

Q17 In Module 3 you learned about *Designing for Context: Messaging & Creative that Resonates.* Please indicate to what extent you agree with the following statements:

	Strongly disagree (1)	Disagree (2)	Neutral (3)	Agree (4)	Strongly agree (5)
The material was new to me (1)	○	○	○	○	○
The module was easy to understand (3)	○	○	○	○	○
The course material was relevant to my work (4)	○	○	○	○	○

Page Break

Q18 In Module 4 you learned about *Tactics for Digital Media and Campaign Implementation.* Please indicate to what extent you agree with the following statements:

	Strongly disagree (1)	Disagree (2)	Neutral (3)	Agree (4)	Strongly agree (5)
The material was new to me (1)	○	○	○	○	○
The module was easy to understand (2)	○	○	○	○	○
The course material was relevant to my work (3)	○	○	○	○	○

Page Break

Q19 In Module 5 you learned about *Metrics that Matter – Understanding Impact.* Please indicate to what extent you agree with the following statements:

	Strongly disagree (1)	Disagree (2)	Neutral (3)	Agree (4)	Strongly agree (5)
The material was new to me (1)	○	○	○	○	○
The module was easy to understand (2)	○	○	○	○	○
The course material was relevant to my work (3)	○	○	○	○	○

Page Break

Q20 How satisfied were you with each of the following course elements?

	Extremely dissatisfied (1)	Somewhat dissatisfied (2)	Neither satisfied nor dissatisfied (3)	Somewhat satisfied (4)	Extremely satisfied (5)
Expert videos (1)	○	○	○	○	○
Case studies/Field perspective videos (2)	○	○	○	○	○
Quizzes (3)	○	○	○	○	○
Homework/Assignments (5)	○	○	○	○	○
Peer Review Process (6)	○	○	○	○	○
Coursera Interface (7)	○	○	○	○	○
Pace of the modules and course material (8)	○	○	○	○	○
Availability of interpretation/ translation (9)	○	○	○	○	○

End of Block: Course Evaluation

Start of Block: Access

Q21 How much time did it take you, per module, to complete the course material and assignments?

- ○ 1-2 hours (1)
- ○ 3-4 hours (2)
- ○ 5-6 hours (3)
- ○ More than 6 hours (4)

Page Break

Q22 How much time did it take you to complete the course, from start to finish?

- Less than 1 month (1)
- 1-2 months (2)
- 3-4 months (3)
- 5-6 months (4)

Page Break

Q23 What technologies did you use to take this course? Please select all that apply:

- ▢ Computer / Laptop (1)
- ▢ Phone (2)
- ▢ Tablet (3)
- ▢ Streaming device (e.g., Apple TV, Roku) (4)
- ▢ If other, please list here (5) ____

Page Break

Q24 From where I took the course, the content was easy to load and view:

○ Strongly disagree (1)

○ Somewhat disagree (2)

○ Neither agree nor disagree (3)

○ Somewhat agree (4)

○ Strongly agree (5)

End of Block: Access

Start of Block: Overall Feedback

Q26 How likely are you to recommend this course to a colleague?

○ Highly Unlikely (1)

○ Unlikely (2)

○ Neutral (3)

○ Likely (4)

○ Highly Likely (5)

Page Break

Q25 If you have suggested improvements for the course, or topic areas to include in the future, please describe them below:

____

Page Break

Q27 Will you share your certificate of completion on your LinkedIn page?

- ◯ Yes (1)
- ◯ No (2)
- ◯ NA - I did not earn the certificate (4)
- ◯ NA - I do not have a LinkedIn page (3)

End of Block: Overall Feedback

Start of Block: Practical Application

Q28 Since starting the course, have you applied any of the skills you learned into practice?

- ◯ Yes - I've applied all skills from the course (1)
- ◯ Yes - I've applied some skills from the course (2)
- ◯ No - I have not applied any skills from the course (3)
- ◯ NA - course skills are not relevant to my current position (4)
- ◯ Prefer not to say (5)

Page Break

Q29 Since starting the course, have you applied any of the following skills?

	Never (1)	Not yet, but I plan to (2)	Rarely/Sometimes (3)	Often (4)	Always (5)
I've used behavioral insights to develop social and behavior change communication (SBCC) (1)	○	○	○	○	○
I've developed a strategic brief for a health communications/campaign strategy (2)	○	○	○	○	○
I've used Canva or a similar program to create assets for a digital campaign (3)	○	○	○	○	○
I've used Ads Manager metrics to design a custom target audience on Meta Platforms (i.e. Facebook/Instagram) or other digital media platforms (4)	○	○	○	○	○
I've tested my campaign strategy using A/B testing or Facebook Brand Lift Studies, prior to launch (5)	○	○	○	○	○
I've evaluated my campaign strategy using survey data or administrative (clinical) data to understand its impact on health outcomes (6)	○	○	○	○	○

Page Break

Q30 Since starting the course, have you been invited to any public speaking opportunities (e.g., at a conference, workshop, webinar, etc.) to talk about your SBCC work using digital media?

- ○ Yes (1)
- ○ No (2)
- ○ Prefer not to say (3)

Page Break

Q31 Since starting the course, have you received any awards for SBCC work using digital media?

- Yes (1)
- No (2)
- Prefer not to say (3)

End of Block: Practical Application

Start of Block: FUNDING

Q32 Does your organization allocate internal funding to SBCC activities using digital media?

- Yes (1)
- No (2)
- I don't know (3)

Page Break

Q34 Of the health initiatives or projects you support, roughly what percent of budget is spent on digital media to drive your health outcomes?

○ % (1) ____

○ I don't know (2)

Page Break

Q35 Since starting the course, how many proposals or funding requests do you think your organization has submitted to support SBCC activities using digital media?

- ○ Number of proposals: (4) ____
- ○ I am not involved in funding requests (5)
- ○ Not applicable (6)

End of Block: FUNDING

Start of Block: Upload

Q36 **Optional.** Please upload an example of a) a recent social media post or b) a recent campaign to drive health outcomes that you have been involved with in your organization since starting the course.

End of Block: Upload
