# Supplementary material for: Digital Media for Health Outcomes: Evaluation Study of a Massive Online Open Course
Source: J Med Internet Res. 2026 Jun 25;28:e85016. doi: 10.2196/85016 (PMC13351640; doi:10.2196/85016)
Supplement: Multimedia Appendix 2 [file jmir_v28i1e85016_app2.docx]

**Supplementary Materials** for *Digital Media for Health Outcomes*: Evaluation of a Massive Online Open Course

**Table S1.** Module-specific evaluation.

| **Module** | **The material was new to me (n, %)** | | | **The material was easy to understand (n, %)** | | | **The material was relevant to my work (n, %)** | | |
| --- | --- | --- | --- | --- | --- | --- | --- | --- | --- |
|  | Disagree | Neutral | Agree | Disagree | Neutral | Agree | Disagree | Neutral | Agree |
| Behavioral insights as a foundation **(Module 1)** | 61 (9.6%) | 118 (19%) | 455 (72%) | 8  (1.3%) | 70 (11.0%) | 556 (88%) | 5 (0.79%) | 80 (13%) | 549 (87%) |
| Crafting your communication strategy **(Module 2)** | 65 (10%) | 118 (19%) | 450 (71%) | 8  (1.3%) | 57 (9.00%) | 568 (90%) | 7  (1.1%) | 60 (9.5%) | 566 (89%) |
| Designing for context: messaging and creative **(Module 3)** | 64 (10.1%) | 109 (17.2%) | 460 (72.7%) | 10 (1.6%) | 63 (9.95%) | 560  (89%) | 6 (0.95%) | 61 (9.6%) | 566 (89%) |
| Tactics for digital media and campaign implementation **(Module 4)** | 47 (7.4%) | 108 (17%) | 478 (76%) | 12 (1.9%) | 81 (13%) | 540 (85%) | 8 (1.3%) | 74 (12%) | 551 (87%) |
| Metrics that matter: understanding impact **(Module 5)** | 49 (7.7%) | 87 (14%) | 497 (79%) | 11 (1.7%) | 80 (13%) | 542 (86%) | 9  (1.4%) | 63 (10%) | 561 (89%) |
